# A Protein-Interaction Array Inside a Living Cell**

**DOI:** 10.1002/anie.201209127

**Published:** 2013-03-04

**Authors:** Silke Gandor, Stephanie Reisewitz, Muthukumaran Venkatachalapathy, Giuseppe Arrabito, Martina Reibner, Hendrik Schröder, Katharina Ruf, Christof M Niemeyer, Philippe I H Bastiaens, Leif Dehmelt

**Affiliations:** Department of Systemic Cell Biology, Max Planck Institute for Molecular PhysiologyOtto-Hahn-Straße 11, 44227 Dortmund (Germany)Chemische Biologie, Technische Universität DortmundOtto-Hahn-Straße 6, 44227 Dortmund (Germany) E-mail: philippe.bastiaens@mpi-dortmund.mpg.deleif.dehmelt@mpi-dortmund.mpg.de; Biologisch-Chemische Mikrostrukturtechnik, Technische Universität DortmundOtto-Hahn-Straße 6, 44227 Dortmund (Germany); Scuola Superiore di CataniaVia Valdisavoia 9, 95123 Catania (Italy); Institute for Biological Interfaces (IBG 1), Karlsruher Institut für Technologie (KIT), Hermann-von-Helmholtz-Platz76344 Eggenstein-Leopoldshafen (Germany)

**Keywords:** multiplexed assay, protein-protein interactions, receptors, signal transduction, surface-immobilization

Cell phenotype is determined by protein network states that are maintained by the dynamics of multiple protein interactions.^1^ Fluorescence microscopy approaches that measure protein interactions in individual cells, such as by Förster resonant energy transfer (FRET), are limited by the spectral separation of fluorophores and thus are most suitable to analyze a single protein interaction in a given cell. However, analysis of correlations between multiple protein interactions is required to uncover the interdependence of protein reactions in dynamic signal networks. Available protein-array technologies enable the parallel analysis of interacting proteins from cell extracts, however, they can only provide a single snapshot of dynamic interaction networks. Moreover, because of the high level of variance from cell to cell in protein expression levels and reaction state, cell extracts only provide an average measure of protein interaction states and therefore the detection of the relations between proteins is blurred. As an intermediate step, a visual immunoprecipitation assay was developed that allowed direct observation of multiple, dynamic protein interactions on immobilized, distinguishable beads in cell extracts.^2^ A microstructuring approach allowed for analysis of the interaction of one naturally occurring receptor type with one of its interaction partners inside cells.^3^ To analyze multiple protein interactions inside a single living cell, multiple receptors must be arranged in a defined pattern to distinguish their identity. Herein, we developed a general strategy to generate protein arrays with multiple arbitrary bait proteins by way of artificial-receptor constructs at sub-cellular feature size and applied this technology to simultaneously measure two-protein interaction kinetics inside an individual living cell.

Protein arrays inside living cells were generated by artificial receptors that transfer a micrometer-scale antibody surface pattern into an ordered array of bait proteins in the plasma membrane (Scheme 1 a). We termed these receptors bait-presenting artificial receptor constructs (bait-PARCs). Bait-PARCs are composed of an intracellular domain that contains an arbitrary bait protein, a single transmembrane domain, and an extracellular domain that contains a viral epitope that directs bait-PARCs towards patterns of cognate immobilized antibodies. Four repeats of the Titin Ig domain I27, act as a spacer to facilitate the interaction of bait-PARCs with the immobilized antibody. The bait-PARCs and the immobilized antibodies do not interact with cellular signaling pathways and therefore minimally perturb cellular function. The prey is expressed in the cytosol as a fluorescent fusion protein. The interaction between multiple, distinct bait proteins on the bait-PARCs with the prey is monitored in living cells using the co-localization of fluorescence signals within an exponentially decaying evanescent field of 50–300 nm depth using total internal reflection fluorescence microscopy (TIRFM). The identity of the bait is determined by the position within the spatial pattern of immobilized antibodies to which the corresponding bait-PARC is recruited.

**Scheme 1:**
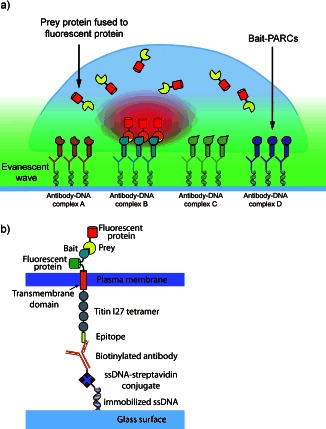
Protein arrays inside living cells. a) Application of bait-presenting artificial receptor constructs (bait-PARCs) to transfer an antibody surface pattern into an ordered array of intracellular bait proteins. b) Schematic illustration of a bait-PARC and cognate immobilized antibody.

To create a pattern of bait-PARCs inside cells, we used DNA-directed immobilization (DDI)^4^ to generate micrometer-scale arrays of antibodies with binding specificity for the peptide epitope on the bait-PARC. The DDI method takes advantage of specific hybridization of complementary oligonucleotides and thereby allows the site-specific capture of sensitive biomolecules by the DNA microstructures on a solid substrate under mild conditions.^5^ Furthermore, the DDI strategy allowed for very flexible surface chemistry in the first micropatterning step, in which chemically stable capture-oligonucleotides were covalently linked to activated glass surfaces using dip-pen nanolithography (DPN).^6^ Oligonucleotides complementary to the immobilized capture-oligonucleotides were covalently linked to streptavidin, and the resulting conjugates were functionalized with biotinylated antibodies and fluorophores. These streptavidin–antibody complexes then bind to the immobilized capture-oligonucleotide arrays. The high specificity of the interaction between complementary DNA oligonucleotide pairs enables the generation of multifunctional antibody arrays (Figure 1 a).

**Figure 1:**
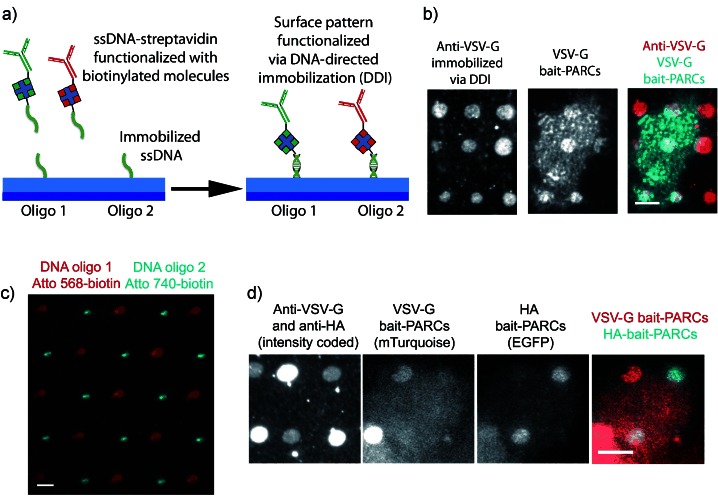
Micropatterning of bait proteins in living cells. a) DDI to generate arrays of immobilized antibodies. b) Bait-PARCs displaying VSV-G epitope tags are recruited to anti-VSV-G functionalized surface patterns within the plasma membrane of COS7 cells. Scale bar=5 μm. c) Selective surface functionalization by DPN and DDI. Scale bar=5 μm. d) Checkerboard patterns of two distinct antibodies, anti-VSV-G and anti-HA, generated by DPN and DDI. Two distinct bait-PARCs, which display the corresponding peptide epitope tags (HA and VSV-G) in their extracellular region were co-expressed in COS7 cells and enriched in the cognate antibody microstructures. Scale bar=10 μm.

First, arrays of a single antibody were generated with an average feature diameter of 4.5±0.5 μm and average feature distance of 11.4±1.4 μm (Figure 1 b). Bait-PARCs displaying three repeats of the VSV-G epitope were recruited to anti-VSV-G microstructures within the plasma membrane of living cells (265±55 % enrichment of mean bait fluorescence intensity in comparison to non-targeted regions). We next generated sub-cellular feature-size arrays of two distinct antibodies. For this, two distinct capture-oligonucleotides were immobilized on glass surfaces in checkerboard patterns using DPN. We then incubated these surfaces with a mixture of two complementary DNA-linked streptavidin conjugates labeled with spectrally separable biotinylated fluorophores. As shown in Figure 1 c, these two conjugates were selectively directed to their cognate microstructures. In subsequent experiments, we encoded identity of the antibody by varying the intensity of the fluorophore (see Experimental Section in Supporting Information). The left-most panel of Figure 1 d shows the checkerboard pattern of two distinct antibodies: anti-VSV-G (high intensity Atto 740) and anti-HA (low intensity Atto 740). Bait-PARCs displaying either the VSV-G or the HA epitope were enriched in their cognate antibody-functionalized microstructures (289±125 % mean bait fluorescence intensity for VSV-G bait-PARCs (mTurquoise) and 322±127 % for HA bait-PARCs (EGFP), compared to non-targeted regions). This shows that the spatially encoded information of an array of surface-linked antibodies can be transferred into an intracellular protein array using bait-PARCs.

Agonist-induced activation of G-protein-coupled receptors (GPCRs) leads to the dissociation of the regulatory and catalytic subunits of protein kinase A (PKA)^7^ by way of a non-linear, allosteric interaction of the second messenger cAMP with two bindings sites on the regulatory subunits. We used this well-established signaling response to validate our approach to study protein interactions in living cells using bait-PARCs. The regulatory subunit II-β (RII-β) was used as the bait and fused to the intracellular region of bait-PARCs displaying VSV-G epitopes (Figure 2 a). We termed these RII-β presenting artificial receptor constructs VSV-G RII-β-PARC. The cytoplasmic catalytic subunit cat-α of PKA was fused to the fluorescent protein mCherry and acts as prey (mCherry-cat-α). As shown in Figure 2 b, the cytosolic prey protein was recruited to bait-PARC-enriched microstructures in resting cells, which contain low levels of cAMP.^8^ Within seconds upon addition of the β-adrenergic receptor agonist isoproterenol, the interaction between bait and prey was lost (Figure 2 b). This shows that activation of these G-protein-coupled receptors increases intracellular cAMP levels, which causes the dissociation of the catalytic subunits from regulatory subunits on bait-PARCs. Interestingly, the dissociation of the bait and prey exhibited an adaptive response, presumably owing to receptor desensitization and hydrolysis of cAMP by phosphodiesterases.^9^ The β-adrenergic receptor antagonist, propranolol, further increased the bait/prey interaction. Subsequent receptor stimulation using isoproterenol could not reactivate the system. However, direct and maximal elevation of intracellular cAMP levels by pharmacological stimulation of adenylate cyclase and inhibition of phosphodiesterase using forskolin/IBMX (F/I) lead to a strong and persistent dissociation of the catalytic and regulatory subunits. This effect was fully reversible following drug washout. In subsequent experiments, this pharmacological stimulation was used to assure that the core components of the cAMP system were functional and to determine the dynamic range of the bait-PARC sensor systems.

**Figure 2:**
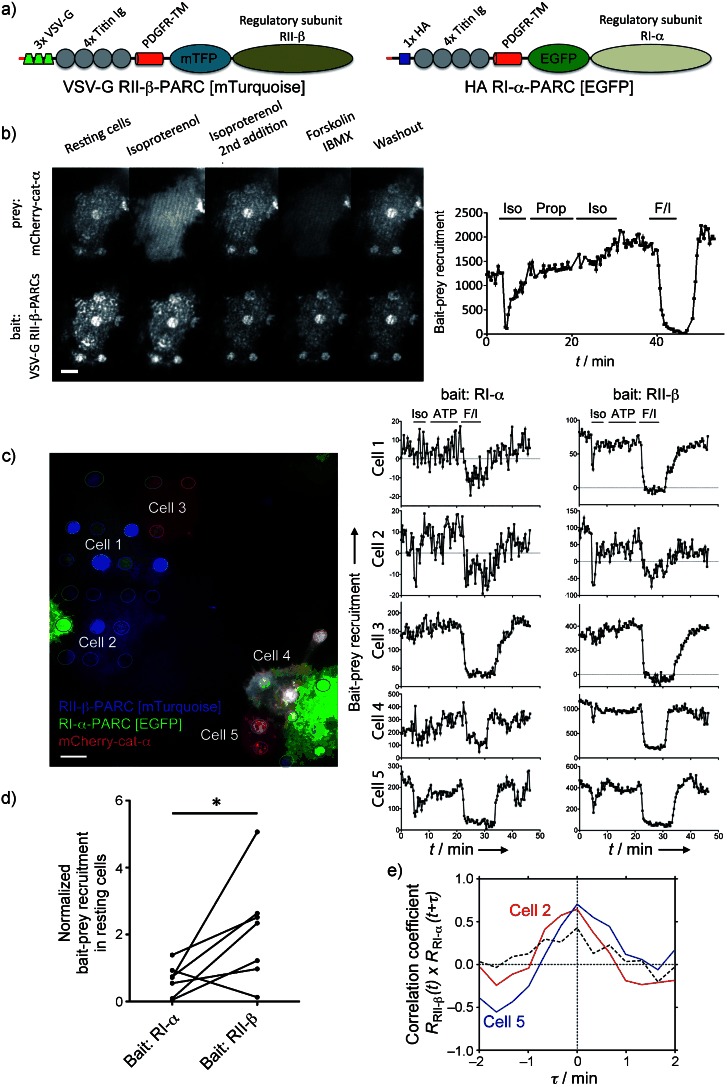
Monitoring protein reaction dynamics inside individual cells. a) Domain structures of bait-PARCs to measure PKA subunit interaction. b) A bait-PARC containing the regulatory domain RII-β of PKA was co-expressed with the cytosolic prey protein (mCherry-cat-α) to monitor their interaction dynamics inside living cells. Left: Recruitment of the prey to bait microstructures before and after pharmacological perturbation. Right: Derived prey recruitment kinetics. Scale bar=5 μm. c) Two distinct regulatory domains on bait-PARCs were co-expressed together with the prey protein mCherry-cat-α. Left: Image of a representative experiment depicting cells grown on a DNA-immobilized antibody array. The checkerboard pattern of antibodies is overlayed with magenta (anti-HA) or cyan (anti-VSVG) circles. Scale bar=10 μm. Right: Derived prey-recruitment kinetics to the two distinct bait proteins. d) Paired measurements of the interaction between the prey protein and the two bait proteins in resting cells. The two experimental groups are significantly different (*p*<0.05; Wilcoxon signed rank test, *n*=7 cells from 3 independent experiments). e) Temporal cross-correlation profiles for the response of the two distinct regulatory subunits during β-adrenergic receptor stimulation. The cross-correlation is calculated from the recruitment kinetics and plotted as a function of the time shift *τ*. The red and blue lines show correlation profiles for two individual cells, the black line shows the average profile of 7 cells. Iso=Isoproterenol; Prop=Propranolol; F/I=forskolin+IBMX; ATP=adenosine triphosphate.

To demonstrate that the dynamics of two distinct protein interactions can be monitored in single cells, two bait-PARCs were generated that were fused to bait proteins with distinct response properties: the regulatory subunits RI-α and RII-β. Each bait-PARC also displayed distinct peptide antigens that are recognized by two corresponding, immobilized antibodies and they were fused to spectrally separable fluorescent proteins (Figure 2 a). As shown in Figure 2 c, the cytosolic prey protein mCherry-cat-α interacts with both bait-PARCs in resting cells. Normalization of prey recruitment to the enrichment of bait proteins allowed direct comparison of the cAMP-dependent regulation of interactions between mCherry-cat-α and the regulatory subunits RI-α and RII-β in individual cells. This key feature enables the identification of relations between these distinct protein interactions. Indeed, we found that mCherry-cat-α bound preferentially to RII-β in resting cells (Figure 2 c,d). This difference cannot be explained by the affinities of the prey to its two alternative bait proteins, as those are very similar (*K*_D_ for RI-α: 0.19 nm; RII-β: 0.6 nm^10^). However, the effective concentration of cAMP to dissociate the cat-α/RI-α interaction is lower than for the cat-α/RII-β interaction (EC_50_ for RI-α: 101 nm; RII-β: 610 nm^10^). Thus, low basal levels of cAMP near the plasma membrane^8^ could partially dissociate the cat-α/RI-α interaction while the cat-α/RII-β interaction remains unaffected.

Simultaneous monitoring of the response profiles of the two distinct bait–prey interactions also enabled analysis of their temporal correlation. In selected individual cells, we observed a clear positive temporal cross-correlation for the cat-α/RI-α and the cat-α/RII-β interaction responses to β-adrenergic receptor stimulation. However, the average cross-correlation from several cells was much weaker (Figure 2 e), showing that single-cell approaches such as the one presented herein are useful to detect relations between proteins that vary in individual cells. Treatment with forskolin/IBMX always strongly and reversibly dissociated the interaction between the catalytic subunit and both regulatory PKA subunits, demonstrating that they are still functional, while only a subset of cells responded to β-adrenergic receptor stimulation (Figure 2 c). This high level of cell-to-cell variance can be explained by adaptive mechanisms in the underlying signal networks.^9^ Because of this variance, relations in response properties between the regulatory subunits, such as their interaction efficiency in resting cells (Figure 2 d) or their temporal cross-correlation profiles (Figure 2 e), are blurred in measurements averaged from many cells. This is overcome by measuring the dynamic response profiles of the interaction between the catalytic and the two distinct regulatory subunits simultaneously in individual cells.

The presented method could be extended to study more than two interactions in parallel. In practice, the number of distinct protein-interaction pairs is limited only by the orthogonality and affinity of the interaction between bait-PARCs and immobilized antibodies and the density of the patterned structures. The antibody–antigen interaction employed in this study is highly specific and many high-affinity antibodies that recognize peptide antigens can be generated.^11^

In summary, we presented the development of a modular protein array to simultaneously measure multiple interactions between bait and prey proteins inside a living cell. We implemented this system to study GPCR signaling dynamics and performed direct simultaneous measurements of two-protein interaction kinetics using bait-PARC arrays with high dynamic range and sensitivity. This enabled the identification of relations between proteins in individual cells. The modular design principle of bait-PARCs enables further extension of the number of simultaneous protein-interaction measurements and thereby allows for the direct analysis of relations between proteins in interconnected signaling networks. This is necessary to unravel mechanisms of signal crosstalk, for example in growth factor signaling, which are otherwise blurred by cell-to-cell variance.

## supplementary-material

As a service to our authors and readers, this journal provides supporting information supplied by the authors. Such materials are peer reviewed and may be re-organized for online delivery, but are not copy-edited or typeset. Technical support issues arising from supporting information (other than missing files) should be addressed to the authors.
