# Supplementary material for: A Protein-Interaction Array Inside a Living Cell
Source: Angew Chem Int Ed Engl. 2013 Mar 4;52(18):4790–4. doi: 10.1002/anie.201209127 (PMC3652028; doi:10.1002/anie.201209127)
Supplement: Supplementary file 1 [file anie0052-4790-SD1.pdf]

Angewandte Chemie
Eine Zeitschrift der Gesellschaft Deutscher Chemiker

Supporting Information

© Wiley-VCH 2013

69451 Weinheim, Germany

A Protein-Interaction Array Inside a Living Cell**

Silke Gandor, Stephanie Reisewitz, Muthukumaran Venkatachalapathy, Giuseppe Arrabito, Martina Reibner, Hendrik Schröder, Katharina Ruf, Christof M. Niemeyer, Philippe I. H. Bastiaens, and Leif Dehmelt**

anie_201209127_sm_miscellaneous_information.pdf

Supporting Information

Content

1. Supporting Schemes

2. Experimental Section

1. Supporting Schemes

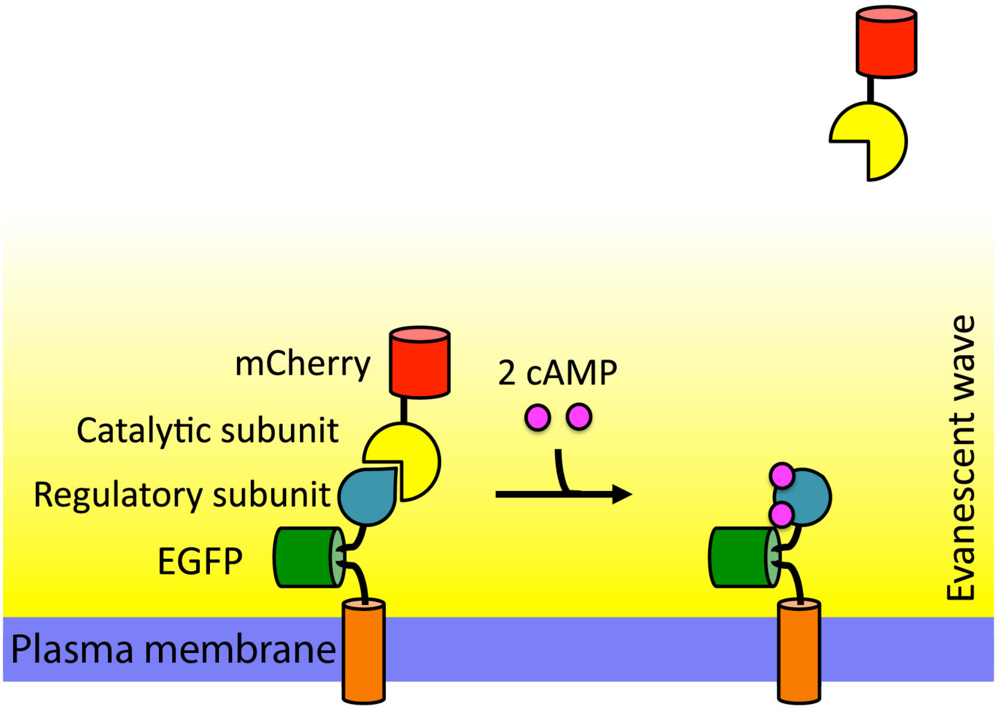

Supporting Scheme 1. Concept of a bait-PARC based sensor for measuring the interaction between PKA subunits. The regulatory subunit is presented as bait and the catalytic subunit acts as prey. In resting cells, bait and prey form a complex. After stimulation of GPCR signalling, cAMP binds to the regulatory subunit causing the dissociation of the RII-β/cat-α complex.

2. Experimental Section

Plasmid Construction

Plasmids coding for HA RI-α-PARC (EGFP) and VSVG RII-β-PARC (mTurquoise) were based on the pDisplay vectors (Invitrogen), which contain the IgK secretion signal peptide and the PDGF transmembrane domain. The stop codon after the transmembrane domain of pDisplay was substituted by NheI and NotI sites encoded on PCR primers and the fluorescent proteins monomeric EGFP (A206K) or mTurquoise (kind gift from Theodorus Gadella, University of Amsterdam) were inserted into the intracellular receptor region via NheI and NotI. The HA epitope (sequence: YPYDVPDYA) is located directly after the secretion signal peptide in pDisplay. In some receptors this epitope was replaced by the VSVG epitope (sequence: YTDIEMNRLGK) encoded on PCR primers via the HindIII and ApaI sites. To improve recruitment of VSVG RII-β-PARC (mTurquoise), 2 additional repeats of the VSVG epitope tag were inserted via annealed oligonucleotide linkers between the BglII and ApaI restriction sites.

Analogous addition of 2 additional HA epitope repeats into HA bait-PARCs inhibited targeting to anti-HA spots, thus, only one repeat is present in that receptor type. Four repeats of the titin IgG domain, which act as a spacer between the transmembrane domain and the epitope tags, were amplified via PCR from the plasmid (I27)4 pQE80L (kind gift of Dr. Julio Fernandez, Columbia University, New York) and inserted between the restriction sites BglII and SalI. Plasmids, which served as templates for amplifying the bait proteins RI-α and RII-β and the prey protein cat-α, were obtained from Susan Taylor (UCSD). The bait proteins were cloned into bait-PARCs via the NotI/XhoI restriction sites and plasmids coding for inducible expression of mCherry-cat-α were generated by inserting the mCherry-cat-α sequence from pcDNA3-pmCherry-PKA-Catα (Susan Taylor) into the plasmid pTRE3G-IRES via the sites SalI and BamHI (the IRES sequence is removed in this cloning step).

Surface chemistry

Microscope coverslips (18x18 mm) were cleaned with abs. EtOH and subsequently functionalized with primary amino groups using 3-aminopropyltriethoxy-silane solution (APTS, Sigma-Aldrich) following previously reported protocols.[1] The amino-silylated slides were washed with EtOH and acetone, dried for 15 min at 110° C and stored at -20° C over night. Subsequently, the activated coverslips were coated with PBAG (Sigma-Aldrich) 1:1 in acetone (w/w) over night, washed with acetone and dried under nitrogen. The coverslips were stored dry at -20° C until use. Attachment of alkylamino-modified capture oligonucleotides (acF5: 5'-CTT ATC GCT TTA TGA CCG GAC C-3'; acF1: 5'-GTA CTT CCT TAA ACG ACG CAG G-3'; acF10: 5'-CGT GTA GCC TTT GTA TTC GTC C-3', each modified with 5' amino link (C6)) was conducted by dip-pen nanolithography, as described below. The resulting slides were incubated overnight and subsequently mounted under a culture dish (BD Falcon™ 35 mm) with a centered hole of 10 mm diameter.

DPN patterning of capture oligonucleotides

All experiments were performed by using a commercial DPN instrument (Nscriptor, Nanoink Inc., USA). Arrays were designed using InkCAD™ software 3.7.1. DPN writing was carried out with the 1D type M passive DPN Pen array (NanoInk, Inc., USA), consisting of 12 silicon nitride tips spaced by 66 µm, each having a 0.6 N/m spring constant. Tips were plasma cleaned (Flecto 10 USB instrument, Plasma Technology GmbH, Germany) before each experiment (O_2 gas, 30-40 s, 50 W, 0.5 mbar) in order to remove organic contaminants and to increase the density of hydroxyl groups on their surface. The hydrophilic tips were then immersed in ink by using the microfluidic ink delivery system, which was also plasma cleaned with O_2 gas (30-40 s, 5 W, 0.5 mbar) to increase the hydrophilicity of the ink channels. The inkwells were filled with the capture-oligonucleotide solution (100 µM, 0.3 µL per well, 30 % v/v glycerol, 30 % w/v PEG1000). Tips were ink loaded by dipping for 5 - 10 s in the end part of the 6 µm wide micro-channels of the inkwell substrate. Excess ink was removed from freshly coated tips by executing

bleeding spots in a non-essential area of the slide prior to the printing of arrays. All patterning experiments were carried out at room temperature and 30 % relative humidity using a dwell time of 0.01 s. Dual oligonucleotide arrays were generated by 1D pen array translation and micrometer-scale alignment with the aid of the alignment software tool of the Nscriptor software.

Preparation of protein arrays

Synthesis and purification of the covalent DNA-STV conjugates (F1, F5 and F10) was carried out using thiolated oligonucleotides (tF1: 5'-CCT GCG TCG TTT AAG GAA GTA C-3'; tF5: 5'-GGT CCG GTC ATA AAG CGA TAA G-3' and tF10: 5'-GGA CGA ATA CAA AGG CTA CAC G-3', each modified with 5' thio link (C6)) and streptavidin (STV), as previously described. [2] In brief, STV (10 nmol) was derivatized with maleimido-groups using the heterobispecific crosslinker (sulfo-SMCC, Pierce), reacted with the thiolated oligonucleotide (10 nmol) and subsequently purified by anion-exchange chromatography. The one-to-one molar ratio of oligonucleotide and STV of the conjugates was verified by gel-electrophoresis and photometric analysis, and the concentration was determined by absorbance measurements. Coupling of the F1, F5 and F10 DNA-STV conjugates with biotinylated antibodies, biotin-anti-VSVG (Abcam, Cambridge, UK) and biotin-anti-HA (Enzo, New York, USA), was achieved by mixing 0.01 mM stock solution of the conjugate in buffer A (10 mM Tris buffer, pH 7.4, 1 mM EDTA) and equimolar amounts (0.01 mM stock solution) of the biotinylated antibodies in buffer B (150 mM NaCl, 3.5 mM NaH_2PO_4, 8 mM Na_2HPO_4, pH 7.3), and 15 minute incubation at room temperature. The prepared conjugates were diluted 1:1 (v/v) with 5 µM Atto 740-biotin (Atto-Tec) or Atto 565-biotin (Atto-Tec) in buffer B and incubated for an additional 15 min. Finally the mixture was diluted to 1:1 (v/v) with buffer C (20 mM Tris buffer, pH 7.5, 150 mM NaCl, 5 mM EDTA, 0.05 % (w/v) Tween-20, 800 µM biotin). Spots functionalized with anti-VSVG-containing conjugates consistently incorporated stronger Atto 740 fluorescence compared to anti-HA-containing conjugates. This difference was used in some experiments to encode antibody identity. Following incubation for another 30 minutes, the DNA-STV-IgG conjugates were mixed to a final concentration of 250 nM each in buffer C.

To reduce non-specific binding of reagents and cells to the glass substrate, the DNA microarray culture dish was pretreated for 30 min with buffer D (20 mM Tris-Cl, 150 mM NaCl, 4.5% (w/v) milk powder, 0.2% (w/v) NaN_3, 5 mM EDTA, 1 mg/ml herring sperm DNA, pH 7.35) and subsequently washed for 15 min with buffer C. To translate the DNA arrays into antibody arrays, 50 µl of the above-described DNA-STV-IgG conjugate solution (250 nM) were then applied to the slide and allowed to hybridize for 60 minutes at room temperature in a humidity chamber (VWR). The microarray culture dish was washed with 2 ml sterile buffer B and incubated with 100 µL laminin (2 µg/ml) for 30 min at 37°C.

Cell culture

COS7 cells were maintained using standard conditions. Co-transfection of plasmids coding for bait-PARCs, tet-inducible mCherry-cat-α and the transactivator pCMV-Tet3G (Clontech) was performed using X-treme Gene 9 (Roche). After transfection, cells were cultured at 25°C to slow down expression and to enhance folding of bait-PARCs. 3 days later, cells were detached after 15 min incubation with 10 mM EDTA (in PBS, pH 7.4). EGFP and mTurquoise positive cells were selected by FACS sorting (FACS Aria, BD Biosciences, Heidelberg, Germany) and re-plated onto antibody-functionalized glass coverslips. Approximately 6-7 h after plating, expression of mCherry-cat-α was induced by overnight addition of 1 μM doxycycline (Clontech). TIRF microscopy was performed at 25° C in HEPES-stabilized imaging medium. Pharmacological manipulation using 10 μM isoproterenol, 10 μM propranolol, 25 μM forskolin, 100 μM IBMX or 10 mM ATP (Sigma-Aldrich) were performed on the microscope stage.

Microscopy

For TIRF microscopy, an Olympus IX-81 microscope equipped with a PlanAPO60xOil TIRFM objective (NA=1.45) was used. For measurements in TIRF mode, a triple bandpass dichroic mirror (U-M3TIR405/488/561, Olympus) was combined with Semrock Brightline emission filters (HC 520/35 and HC 629/53, AHF Analysentechnik, Tübingen, Germany), the 488 nm line of a 400 mW Argon ion laser (model # 543-A-A03, Melles Griot, Bensheim, Germany), a 405 nm, 50 mW and a 561 nm, 100 mW CellR diode laser (Olympus). Wide-field images of Atto 740 labeled micropatterns were obtained on the same microscope via illumination with the MT-20 device (Olympus) and the Chroma ET-Cy7 filter set (excitation: ET710/75x; emission: ET810/90m; beamsplitter: T760lpxr; AHF Analysentechnik, Tübingen, Germany). This combination of excitation/emission filters allowed complete spectral separation of mTurquoise (TFP), EGFP, mCherry and Atto 740 fluorophores. For detection, an EMCCD camera (C9100-13, Hamamatsu, Hersching, Germany) was used. All microscope components were controlled by the CellR software (Olympus).

Data Analysis

Image analysis and image processing was performed using ImageJ (NIH, Bethesda). Image manipulations were limited to cropping, scaling, rotation, adjustment of levels and addition of clearly identifiable labels or symbols. The levels in all image panels in time series are adjusted identically. To correct for lateral drift in the stage position, an image stabilizer plugin for ImageJ was used (K. Li, "The image stabilizer plugin for ImageJ," http://www.cs.cmu.edu/~kangli/code/Image_Stabilizer.html, February, 2008). The average fluorescence intensity of the bait and prey proteins was measured in circular regions using the Intensity vs Time Monitor function in ImageJ. Background corrected measurements in microstructured spots (I_{spot}) and cellular background levels next to the

spots I_{cells} were obtained by subtracting background levels outside cells (I_{bg}). Fluctuations in excitation intensity during time-lapse recordings were corrected by dividing intensities from individual frames by the corresponding background levels I_{bg} and multiplying with average background levels. The relative enrichment of bait proteins in microstructured spots was calculated as the ratio between the background corrected intensities in spots vs next to the spots: relative enrichment (in %)=100* I_{spot} / I_{cells}. The recruitment of prey to bait proteins was calculated as follows: The intensity $I_{bait,\ spot}$ corresponds to the sum of fluorescence intensities originating from bait-PARCs that are bound to micropatterned spots and freely diffusing bait-PARCs ($I_{bait,\ spot} = I_{bait,immobilized} + I_{bait,free}$). The intensity $I_{bait,\ cells}$ originates only from freely diffusing bait-PARCs ($I_{bait,\ cells} = I_{bait,free}$). Thus, the difference $I_{bait,\ spot} - I_{bait,\ cell}$ is a measure for the intensity of bait protein immobilized to microstructured spots ($I_{bait,immobilized} = I_{bait,\ spot} - I_{bait,\ cell}$). To yield a measure that is independent of total fluorescence intensities, $I_{bait,immobilized}$ was normalized by dividing with the total fluorescence intensity ($I_{bait,\ total} = I_{bait,\ spot}$). This normalized measure was termed the enrichment factor for bait proteins $E_{bait} = I_{bait,immobilized}/I_{bait,\ total}$. The enrichment factor E_{bait} is 1, if bait-PARCs are completely localized to the micropatterned spots, i.e. if all intensity from the bait-PARCs is in the spot area and no intensity is outside the spot area. The enrichment factor E_{bait} is 0, if the intensity is equal inside and outside the spot. The intensity $I_{prey,\ spot}$ corresponds to the sum of fluorescence intensities originating from prey molecules bound to immobilized bait-PARCs ($I_{prey,\ bound}$), those bound to freely diffusing bait-PARCs ($I_{prey,\ bound*}$), and freely diffusing cytosolic prey molecules excited in the TIRF field ($I_{prey,\ cytosolic}$): $I_{prey,\ spot} = I_{prey,\ bound} + I_{prey,\ bound*} + I_{prey,\ cytosolic}$ .The intensity $I_{prey,\ cells}$ originates only from prey molecules bound to freely diffusing bait-PARCs and freely diffusing cytosolic prey molecules: $I_{prey,\ cells} = I_{prey,\ bound*} + I_{prey,\ cytosolic}$. Thus, the difference $I_{prey,\ spot} – I_{prey,\ cell}$ is a measure for the intensity of prey protein bound to immobilized bait-PARCs ($I_{prey,\ bound} = I_{prey,\ spot} – I_{prey,\ cell}$). $I_{prey,bound}$ is dependent on the enrichment factor of the bait E_{bait}, i.e. if the bait presenting artificial receptors are only partially enriched in antibody microstructures, a smaller value for $I_{prey,\ bound}$ is measured. Thus, to measure, how efficiently prey molecules are recruited to bait-PARCs (the bait-prey recruitment R), $I_{prey,bound}$ was divided by the bait enrichment factor (E_{bait}): (R = $I_{prey,bound}/E_{bait}$). The recruitment R is independent of the level of bait enrichment and can therefore be used to compare multiple bait/prey interaction pairs in an individual cell. To compare the recruitment between different cells (Figure 3d), it was additionally normalized to prey fluorescence intensity levels $I_{prey,\ cells}$. Statistical analysis of the normalized recruitment of the prey mCherry cat-α to either of the two bait-PARCs: HA RI-α-PARC (EGFP) and VSVG RII-β-PARC (mTurquoise), was performed with GraphPad Prism 5.0 (Statcon, Witzenhausen, Germany). Cross-correlation analysis was performed using Excel (Microsoft Deutschland GmbH), by calculating Pearson's r for the recruitment kinetics of RII-β and the recruitment kinetics of RI-α shifted by consecutive single time steps.

References

[1] S. Reisewitz, H. Schroeder, N. Tort, K. A. Edwards, A. J. Baeumner, C. M. Niemeyer, *Small* **2010**, *6*, 2162-2168.

[2] R. Wacker, C. M. Niemeyer, *Current Protocols in Nucleic Acid Chemistry* **2005**, Unit 12.17
